# Biocompatible and antibacterial *Flammulina velutipes*-based natural hybrid cryogel to treat noncompressible hemorrhages and skin defects

**DOI:** 10.3389/fbioe.2022.960407

**Published:** 2022-10-11

**Authors:** Yufan Zhu, Feixiang Chen, Minhao Wu, Jieyu Xiang, Feifei Yan, Yuanlong Xie, Zan Tong, Yun Chen, Lin Cai

**Affiliations:** ^1^ Department of Spine Surgery and Musculoskeletal Tumor, Zhongnan Hospital of Wuhan University, Wuhan, China; ^2^ Department of Biomedical Engineering and Hubei Province Key Laboratory of Allergy and Immune Related Diseases, School of Basic Medical Sciences, Wuhan University, Wuhan, China; ^3^ Department of Plastic Surgery, Zhongnan Hospital of Wuhan University, Wuhan, China

**Keywords:** *Flammulina velutipes* polysaccharides, hydroxyethyl cellulose, cryogel, wound healing, hemostasis

## Abstract

Hemorrhage, infection, and frequent replacement of dressings bring great clinical challenges to wound healing. In this work, *Flammulina velutipes* extract (FV) and hydroxyethyl cellulose (HEC) were chemically cross-linked and freeze-dried to obtain novel HFV cryogels (named HFVn, with *n* = 10, 40, or 70 corresponding to the weight percentage of the FV content), which were constructed for wound hemostasis and full-thickness skin defect repair. Systematic characterization experiments were performed to assess the morphology, mechanical properties, hydrophilic properties, and degradation rate of the cryogels. The results indicated that HFV70 showed a loose interconnected-porous structure and exhibited the highest porosity (95%) and water uptake ratio (over 2,500%) with a desirable degradation rate and shape memory properties. *In vitro* cell culture and hemocompatibility experiments indicated that HFV70 showed improved cytocompatibility and hemocompatibility. It can effectively mimic the extracellular matrix microenvironment and support the adhesion and proliferation of L929 cells, and its hemolysis rate *in vitro* was less than 5%. Moreover, HFV70 effectively induced tube formation in HUVEC cells *in vitro*. The results of the bacteriostatic annulus confirmed that HFV70 significantly inhibited the growth of Gram-negative *E. coli* and Gram-positive *S. aureus*. In addition, HFV70 showed ideal antioxidant properties, with the DPPH scavenging rate *in vitro* reaching 74.55%. *In vivo* rat liver hemostasis experiments confirmed that HFV70 showed rapid and effective hemostasis, with effects comparable to those of commercial gelatin sponges. Furthermore, when applied to the repair of full-thickness skin defects in a rat model, HFV70 significantly promoted tissue regeneration. Histological analysis further confirmed the improved pro-angiogenic and anti-inflammatory activity of HFV70 *in vivo*. Collectively, our results demonstrated the potential of HFV70 in the treatment of full-thickness skin defects and rapid hemostasis.

## 1 Introduction

Extensive full-thickness wounds take a long time to repair, and the accompanying blood loss and infection problems are severe clinical challenges (Liu et al., 2019). Rapid closure of the wound site is critical to restoring the barrier function (Chen et al., 2021a). There is strong evidence that wound healing occurs better in a wet or moist environment than that under dry treatment. The improvement can be attributed to a variety of mechanisms, which include faster epithelialization, easier migration of epidermal cells on a moist surface, and the prolonged presence of proteinases and growth factors (Junker et al., 2013). Bioactive scaffolds for skin substitute products play an important role in the process of wound healing, which can temporarily replace the important function of the skin until the wound is healed (Jayakumar et al., 2011; Zhang et al., 2019).

Based on the wet healing theory, hydrogels have advantages in many aspects compared with traditional dry dressings. At the macro level, hydrogels can keep the wound surface moist and temperature-stable, absorb wound exudates, and maintain gas exchange. Also, they are easy to remove from the wound surface, making it painless for patients during the dressing change (Brumberg et al., 2021). At the micro level, hydrogels can closely simulate the mechanical properties of the extracellular matrix (ECM), and in some cases, they also include chemical properties (Pina et al., 2019).

In recent years, hydrogels have been developed and applied extensively, but there are also some disadvantages, such as insufficient mechanical properties and a lack of enough interconnected porosity. The ideal modern biological scaffolds for wound healing should be biocompatible, biodegradable, and non-antigenic and show a 3D structure with high porosity and suitable pore size, which can provide adequate mechanical support, directly promote cell growth and angiogenesis, and act as a carrier for cell metastasis or as a nidus for cell recruitment/migration from the surrounding tissue environment (Razavi et al., 2019). Scaffold porosity permits cell migration and tissue integration, which promote neovascular growth (Ditta et al., 2020). The effect of the implant pore size on tissue regeneration is emphasized by experiments demonstrating the optimum pore size of 20–125 μm for regeneration of the adult mammalian skin (Annabi et al., 2010). The introduction of macropores is an effective way to improve scaffold vascularization. Cryogels are sponge-like improved hydrogel materials obtained by freezing polymerization of the prepolymer at subzero temperatures and using ice crystal as a pore-forming agent (Jonidi Shariatzadeh et al., 2021). The characteristic of an interconnected macroporous structure allows the free water to squeeze out and quickly recover to its original shape by absorbing water (Hou et al., 2020), and such a property is called the water-responsive shape memory. Additionally, the macroporous structure endows the cryogel with high resilience to compressive strain, which enables the cryogel to adjust to its shape upon its application for filling a shape-specific geometric structure/void, which can prevent the collapse of the defect while also controlling bleeding quickly (Zhao et al., 2018). A variety of water-responsive shape memory polymers have been developed based on natural products, such as animal hair (Xiao and Hu, 2016), peacock’s tail covert feather (Liu et al., 2015), and luffa sponges (Shen et al., 2014).

Compared with synthetic chemicals, natural products often have lower costs, better biocompatibility, and better biodegradability. Natural products are selected as ideal raw materials for production under several conditions: they have sufficient sources, low batch variation, and excellent biological activity (Troy et al., 2021). Fungus, as a renewable, cheap resource, has great potential for application in this regard. *Flammulina velutipes* (FV), also called golden needle mushroom or enokitake, is the fourth most popular edible fungus in the world (Jing et al., 2014). Several compounds have been isolated from different parts of FV, including carbohydrates, protein, lipids, glycoproteins, phenols, and sesquiterpenes (Banerjee et al., 2020). Many studies have proved that the main active substances of FV are polysaccharides and glycoprotein, which have a variety of bioactivities, such as anti-oxidation, immune regulation, anti-inflammation, liver protection, anti-tumor effect, anti-hyperlipidemia, improving memory, and resisting decrepitude (Wu et al., 2010; Shi et al., 2012; Wang et al., 2018; Zhang et al., 2018; Chen et al., 2019; Wang and Zhang, 2021). Previously, we demonstrated that the natural FV nerve guide catheter is a potential biomaterial for peripheral nerve regeneration (Chen et al., 2021b). In our previous research, we used FV as raw material and fabricated a novel seedbed-like scaffold by the frozen slicing method. FV polysaccharide-derived scaffolds treated with NaOH have good ductility, antibacterial properties, and biocompatibility and can effectively accelerate full-thickness skin wound healing and hair follicle regeneration (Chen et al., 2021a). However, due to the flat scaffold morphology, which is difficult to cope with hemorrhagic, chronic, prolonged, deep, and exudative wounds due to its insufficient mechanical strength and water retention capacity, the abovementioned drawbacks make it limited in clinical applications.

In view of the abovementioned facts, we developed a green and environmental strategy to fabricate a new multifunctional (hemostatic, antibacterial, antioxidant, and angiogenic properties) shape memory cryogel *via* physical mixing, chemical crosslinking, and freeze-drying. The FV polysaccharide extract and hydroxyethyl cellulose (HEC) solution at different mixing ratios were used to design these hydrogel scaffolds (HFVs). The pure FV polysaccharide extract cannot get a stable and formed cryogel through self-crosslinking, and its product dissolves rapidly when it meets water. Therefore, we introduced HEC that is rich in -OH as the skeleton of cryogels to enhance the mechanical strength of the product and control the degradation rate (Zulkifli et al., 2019; Wu et al., 2020). We evaluated the comprehensive properties of different mixing ratios from their morphology, physical properties, and antioxidant activity. In addition, good cytocompatibility, antibacterial behavior, and pro-angiogenic properties have also been confirmed. Finally, we selected an optimal ratio, which has excellent shape memory performance and biological activity, and applied it to the rat liver defect model and full-thickness skin defect model. All these results indicate that HFVs have the clinical translational capacity to provide effective treatment for the filling and repair of soft tissue defects.

## 2 Materials and methods

### 2.1 Materials

Commercial *Flammulina velutipes* were obtained from the Wuhan Ruyi Edible Fungi Research and Development Center (Wuhan, China). Hydroxyethyl cellulose (HEC), with a viscosity of 30,000 mPa, was purchased from Shandong Head Reagent Co., Ltd. (Shandong, China). Epichlorohydrin (ECH) and sodium hydroxide (NaOH) were purchased from Sinopharm Chemical Reagent Co., Ltd. (Shanghai, China). 1,1-Diphenyl-2-picrylhydrazyl (DPPH) was provided by Sinopharm Chemical Reagent Co., Ltd. (Shanghai, China). All of the chemical reagents used were of analytical grade without further purification.

### 2.2 Preparation of HFV cryogels

#### 2.2.1 FV extraction

The fruiting bodies of fresh *Flammulina velutipes* were washed thoroughly using deionized water. It was then dried by the vacuum freeze dryer before being ground into powder using a grinder through a 200-mesh screen.

#### 2.2.2 HFV cryogel preparation

**FIGURE 1:**
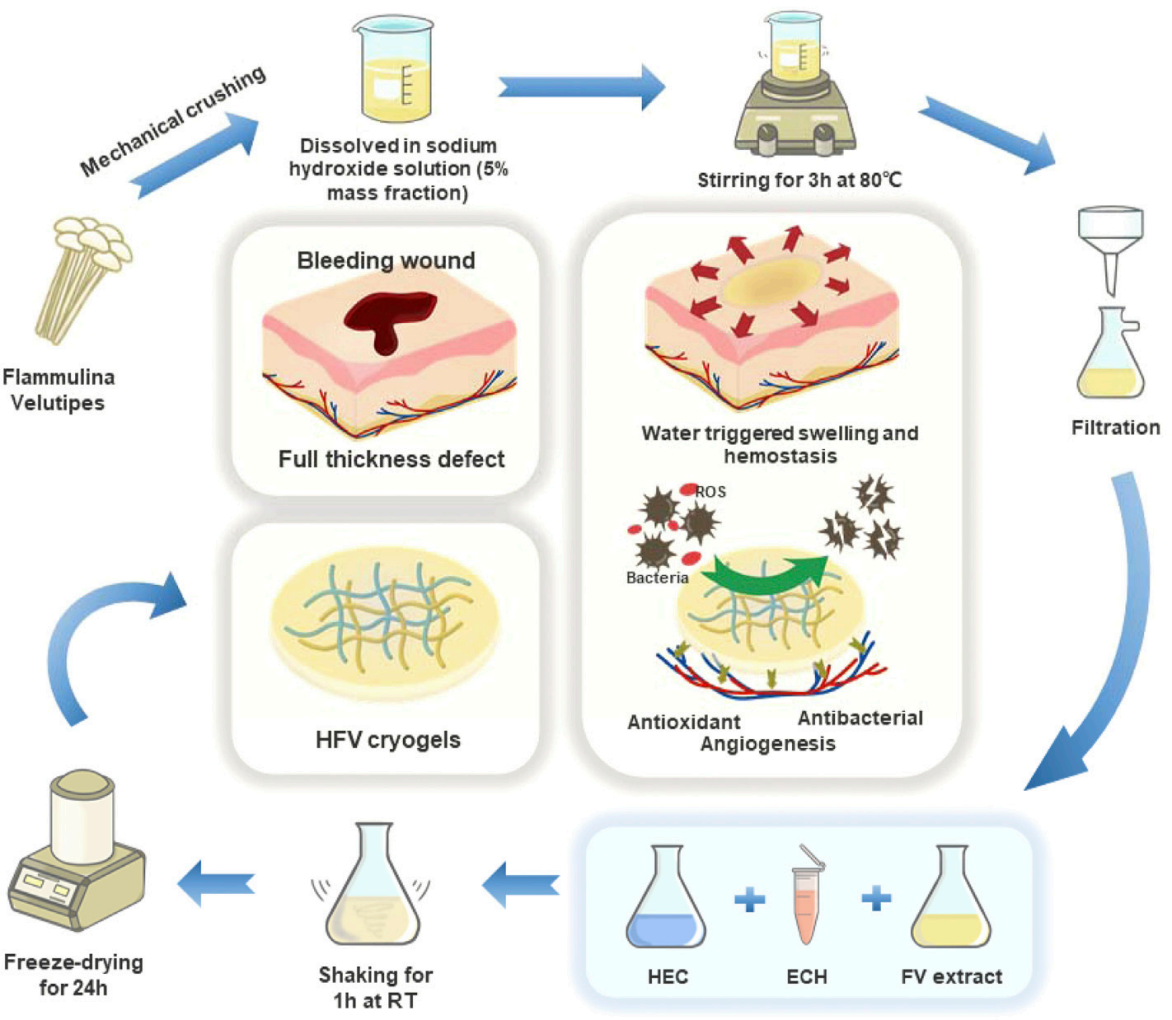
Preparation process and biological function of HFVs.

First, 10 g of the FV powder was weighed and dissolved as a viscous solution with a mass fraction of 10% using 90 g of a 5 wt% NaOH solution (stirring at 80°C for 3 h). Then, 4 g of the HEC powder was added to 196 g of deionized water and allowed to stand for 0.5 h, followed by repeated stirring and standing until it was completely dissolved in deionized water to obtain a 2% HEC solution. The abovementioned 2% HEC solution and 10% FV solution were blended at different ratios, and a crosslinking agent, epichlorohydrin (ECH), was added. The mass of ECH is 20% of the dry weight of the FV/HEC mixture. A clear and transparent blended solution of cross-linked HEC/FV was obtained by mixing with stirring and degassing by centrifugation. The solution was poured into a 100-cm^2^ polystyrene plate and stored at −20°C overnight and then freeze-dried. The dry FV/HEC scaffold was further rinsed using deionized water for 24 h until pH became neutral, followed by freezing and lyophilization at −50°C for 24 h. The cryogel scaffolds were coded as HFVn (*n* = 10, 40, 70 to the respective W_FV_). For *in vitro* and *in vivo* studies, all samples were sterilized *via* ethylene oxide (EO) treatment utilizing an EO sterilizer (Steri-Vac™, 3 M Shanghai, China) for 12 h in sterilization pouches. The preparation process of HFVs is shown in Figure 1.

### 2.3 Biochemical component identification of FV extracts

The biological components of the NaOH extract of FV were identified. The muffle furnace method (Begum, 2014) was used to estimate the ash contents. The Kjeldahl method (Begum, 2014) was used to determine the total protein content. The phenol-vitriolic colorimetry method (Feriani et al., 2020) was used to obtain the total polysaccharide content.

### 2.4 Characterization of HFVs

#### 2.4.1 Macroscopic and microscopic observation

The appearance of dry and wet HFVs was photographed using iPhone 11. High-resolution micro-CT (SkyScan 1276, Bruker, Germany) was utilized to characterize the integral microstructure. The HFVs were freeze-dried and coated with gold. The surface and section structure of the HFVs were observed under a scanning electron microscope (SEM; VEGA3, TESCAN, Czech Republic) with a voltage of 20 kV. Magnification was set at ×300 and ×1500 objectives. The average pore size was quantified based on three SEM images of each scaffold (≥30 pores per image) analyzed with ImageJ software (NIH, Bethesda, MD). The ethanol displacement method was used to determine the porosity of the FVC, as previously described (Liu et al., 2018).

#### 2.4.2 Fourier transform infrared spectroscopy

The HFVs were vacuum-dried and milled into powders. The infrared spectrum of the powder samples was recorded using a Fourier transform infrared spectrometer (TNZ1–5700, Nicolet, United States) within a scanning range of 4,000–400 cm^−1^.

#### 2.4.3 X-ray diffraction

The X-ray diffraction spectrum of the abovementioned samples was determined by using an X-ray diffractometer (D-Advance, Bruker, United States) with a scanning range of 5–55° and a scanning rate of 2°/min. The results were analyzed by MDI Jade 5.0 software.

### 2.5 Mechanical properties

The uniaxial tensile and compression testing of the HFVs was carried out using a Zwick (model Z020) universal mechanical testing machine. The samples for the tensile test have a diameter of 10 mm and a height of 5 mm, and the compression rate is 0.5 mm min^−1^. The samples for the tensile test were soaked with PBS buffer and cut into 20 mm × 10 mm × 5 mm strips, then fixed on the molds, and stretched at a constant speed (5 mm/min) until they broke. Every group was measured three times, and the average value was calculated.

### 2.6 Water absorption and retention capacity

The swelling capacity of HFVs was measured as follows. The samples were freeze-dried sufficiently (weighed as W_0_) and immersed in distilled PBS solution (pH 7.4, 0.1 M) at 37°C. Then, they were taken out at the predestined time and dried superficially with a filter paper, weighed as W_1_. The swelling ratio of HFVs was calculated by equation A.

Equation A:

$$Water\;absorption\;ratio=\frac{W_{1}-W_{0}}{W_{0}}\times 100\%.$$

The water retention ability was tested by weighing samples at pre-determined intervals. The swollen samples were weighed (M_0_) and then placed in a sealed dryer equipped with CaCl_2_. Each sample was taken out at different time points and weighed (M_t_). The water retention ratio of HFVs was calculated by equation B.

Equation B:

$$Water\;retention\;ratio=\frac{M_{t}}{M_{0}}\times 100\%.$$

Each sample was measured three times, and its average value was taken.

### 2.7 *In vitro* degradation test

To investigate the degradation behavior of the HFVs, 1 cm × 1 cm × 1 cm cubic HFVs were sterilized by ^60^Co irradiation (10 kGy) and weighed (M_0_), and then incubated at 37°C in PBS for 14 days. The buffer solution was exchanged every third day. Three samples at each time point (1, 3, 7, 10, and 14 days) were freeze-dried and weighed (M_t_), and the *in vitro* degradation ratio was calculated using equation C.

Equation C:

$$In\;vitro\;degradation\;ratio=\frac{{M_{0}-M}_{t}}{M_{0}}\times 100\%.$$

### 2.8 *In vitro* antioxidant test

The antioxidant efficiency of specimens was tested by measuring their capacity to scavenge the stable 1, 1-diphenyl-2-picrylhydrazyl (DPPH) free radical. We made a minor modification to the method reported by Serpen et al. (2007). HFV samples were crushed into powder by liquid nitrogen treatment; 0.2 g of HFV samples (S) were prepared in 24-well plates and immersed with 1 ml absolute ethanol. Then, 100 μL of DPPH solution (0.5 mM in ethanol) was added and incubated for 15 min in the dark. A measure of 0.2 ml of deionized water was used as the control group (C). The absorbance (A) at 517 nm of the mixture reaction was measured. The DPPH degradation was calculated using equation D.

Equation D:

$$Scavenging\;effect=\frac{A_{c}-A_{s}}{A_{c}}\times 100\%.$$

### 2.9 *In vitro* antibacterial test

The antibacterial activities of the HFVs were tested by the agar diffusion method. Gram-negative *Escherichia coli* (*E. coli*, ATCC 8734) or Gram-positive *Staphylococcus aureus* (*S. aureus*, ATCC 6535) cultures were diluted into 10^5^–10^6^ CFU/ml suspension. Afterward, 200 μL of cultured strains were evenly spread on the plate surface of the LB solid agar plate to get a bacterial lawn. Circular discs of HFVs (5 mm diameter, 1 mm thick) were put on the agar plate in sequence. The filter paper of the same size treated with ampicillin (50 μL/ml) served as the positive control, and a blank filter paper of the same size was used as the negative control. After being cultured overnight, the images and areas of the zone of inhibition (ZOI) were recorded and measured.

### 2.10 *In vitro* biocompatibility evaluations

#### 2.10.1 Cytocompatibility evaluation

Fully freeze-dried HFVs were crushed into powder by liquid nitrogen treatment and then sterilized by ^60^Co irradiation (10 kGy). Sterilized HFV powder was soaked in the complete culture medium mentioned earlier (0.2 g of powder per 1 ml completed culture medium) at 37°C for 72 h. After centrifugation at 1,000 rpm for 5 min and filtration through a 0.22-μm filter (PALL, United States), the supernatants were collected as the extract and stored at 4°C before use. L929 cells were seeded into 96-well tissue culture plates at a density of 1 × 10^3^ cells per well at 37°C with 5% CO_2_ for 24 h. The medium was then discarded and replaced with sample extracts. After incubating for 1, 2, and 3 days, the cells were treated with CCK8. The absorbance values were detected at 450 nm with a plate reader (Multiskan FC, Thermo Scientific).

#### 2.10.2 Morphology observation of cell adhesion

Before being cultured with cells, 0.5 cm × 0.5 cm × 0.5 cm cubic HFVs were sterilized by ^60^Co irradiation (10 kGy). L929 cells were seeded on HFVs at a density of 2 × 10^4^ cells/sample and cultured at 37°C with 5% CO_2_ for 3 days. The samples were fixed by immersion in 2.5% glutaraldehyde for 8 h. After rinsing with PBS, the samples were dehydrated through a graded ethanol series (50, 70, 80, 90, and 100%) for 15 min at each step. Then, they were freeze-dried and coated with gold. Samples were then collected for morphology observation by SEM (SEM; VEGA3, TESCAN, Czech Republic) or by confocal laser scanning (Leica-LCS-SP8-STED, Leica, Germany). For the observation using a confocal laser scanning microscope, the cell nuclei were stained by PI, and the cell membrane was stained by DiO.

#### 2.10.3 Hemocompatibility study

A hemocompatibility study was carried out following a reported protocol (Evans et al., 2013). Packed red blood cells (RBCs) were diluted in saline to obtain 1% (vol/vol) RBC suspension. Negative (N) and positive (P) controls used in this experiment are normal saline and Triton-X 100 (50 μL), respectively. HFV samples (S) were crushed into powder by liquid nitrogen treatment, 0.2 g per sample. Samples were incubated with RBC suspension (1 ml) for 3 h at 37°C. Absorbance (A, OD at 540 nm) of the aliquots after centrifugation of incubated samples (S) was obtained from the microplate reader. Equation E given as follows was used to evaluate hemolysis (%).

Equation E:

$$Hemolysis=\frac{A_{S}-A_{N}}{A_{P}-A_{S}}\times 100\%.$$

#### 2.10.4 Endothelial tube formation assay

For the human umbilical vein endothelial cell (HUVEC) tube formation assay, 1×10^5^ HUVECs were seeded onto the Matrigel films in 24-well plates and treated with 50% HFV extract and 50% ECM supplemented with 2% FBS. Cells were incubated for 6 h and imaged by bright-field microscopy. Tube length was quantified by AngioTool software.

### 2.11 Animal studies

#### 2.11.1 *In vivo* hemostatic test

The hemostatic ability of the HFVs was evaluated by rat liver perforation wound models. Sprague–Dawley (SD) rats (male, weight of 250–300 g, 7–8 weeks) were anesthetized using 1.5% isoflurane and fixed on a surgical corkboard tilted at about 30°. Their abdomens were disinfected with 75% ethanol. Then, their livers were exposed through an abdominal incision and cleaned with normal saline solution, and the abdominal dropsy was cleaned with a piece of gauze. Then, the livers were placed onto the surface of the pre-weighted filter paper. Bleeding of the liver was induced using a puncher with a diameter of 5 mm, and dry HFV70 (diameter of 5 mm, height of 8 mm) was immediately applied to the bleeding site, respectively. The same size commercial gelatin sponge (Xiang’en Medical Technology Development Co., Ltd., Jiangxi Province) was used as a positive control and double-layer gauze as a negative control. After 3 min, the weight of the blood-absorbed filter paper was determined and compared with a blank control group (no treatment after pricking the liver). The hemostatic process was recorded with an iPhone 11 device.

#### 2.11.2 *In vivo* wound healing assay

The effect of HFVs on incision closure was evaluated by a full-thickness rat skin incision model. Forty-eight female Sprague–Dawley (SD) rats (6 weeks old, 180–200 g) were obtained from the Laboratory Animal Center of Wuhan University and randomly divided into four groups with 12 animals in each group: the HFV70 group, chitosan group as the positive control, gauze group as the negative control, and blank control (NC) group. Rats were anesthetized and maintained by 3% isoflurane with a face mask. Then, two 10-mm diameter round full-thickness excisional skin wounds were created on each side of the midline using a biopsy puncher, following the hair removal from the dorsal skin. It is worth noting that a donut-shaped silicone splint was glued to the skin using biomedical glue with the wound centered in the splint. The splint was used to stabilize the wound and avoid contraction, making the wound area comparable. The wound area was recorded (day 0, day 4, day 8, and day 16) with the iPhone 11 device. The body temperature in each group was recorded using an ear thermometer.

The *in vivo* study was conducted according to the guidelines of the Declaration of Helsinki and approved by the Institutional Review Board (or Ethics Committee) of the Institutional Animal Care and Use Committee, Wuhan University Center for Animal Experiment (protocol code WP 2020-08057), on 5 October 2020.

### 2.12 Histological analysis

To evaluate the histological properties of the groups at the metaphase and telophase, on day 8 and day 16, the rats were executed, and the tissues were collected. The regenerated wound tissues plus the surrounding normal area were harvested and fixed in 10% formalin solution. Then, the samples were processed through the spending dehydration (in ethanol solutions), clearance (in xylol), paraffinization (in melted paraffin), and embedding (in paraffin) stages, respectively, followed by sectioning using a microtome to achieve the tissue slices with 5 μm thickness. The obtained slides were stained with hematoxylin and eosin (H&E) and Masson’s trichrome stain. IHC evaluations for CD31 and CK10 (Abcam) markers were conducted with the obtained sections.

### 2.13 Statistical analysis

All experiments were performed, at least, in triplicate, and the obtained results were presented as average ± SD (standard deviation). The standard *t*-test was carried out to compare the statistical significance between the two groups (^*^ *p* < 0.05, ^**^ *p* < 0.01, ^***^ *p* < 0.001, and ^****^ *p* < 0.0001), which was statistically significant.

## 3 Results and discussion

### 3.1 Biochemical component of FV extracts

We identified the biological components of the FV sodium hydroxide extract. As shown in Supplementary Figure S1, the biochemical component of the sample was mainly polysaccharide, whose percentage content reached 59.11 ± 0.90%. The protein content was only second to the polysaccharide, reaching 21.84 ± 0.99%. In our previous study, the polysaccharide content of seedbed-like FV polysaccharide–derived scaffolds was 56.29 ± 0.07%, and the protein content was 24.28 ± 0.21%, which was approximate to the biochemical composition of this study (Chen et al., 2021a). We conjecture that it may be the different treatments (slicing or grinding) on the fruiting bodies of FV before NaOH treatment that caused a little difference in biochemical composition.

### 3.2 Physicochemical characterization of the HFVs

#### 3.2.1 Fourier transform infrared spectroscopy and X-ray diffraction

**FIGURE 2:**
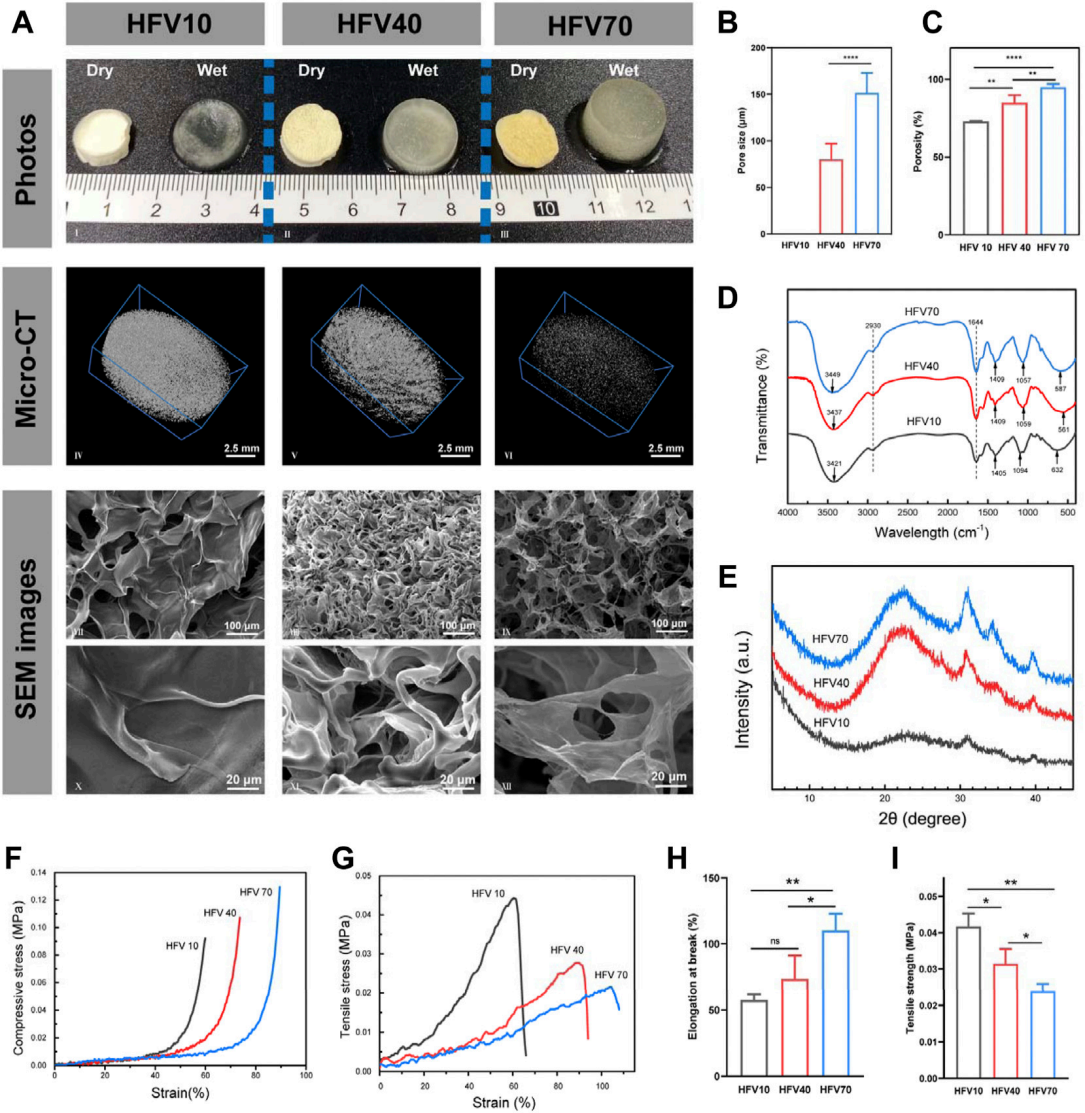
Appearance, physical characterization, and mechanical properties of HFVs. **(A)** Macro and micromorphology of HFVs: photographs (I–III), micro-CT images (IV–VI), and cross-sectional SEM images (VII–IX: low-magnification, 300 ×; X–XII: high magnification, 1500 ×). **(B)** Average pore size of HFVs. **(C)** Porosity of HFVs. **(D)** FTIR spectra. **(E)** XRD patterns. **(F)** Compressive stress–strain curve. **(G)** Tensile stress–strain curve. **(H)** Elongation at the break. **(I)** Tensile strength. *p < 0.05; **p < 0.01.

The FT-IR spectra of HFVs are presented in Figure 2D. From the FT-IR spectrum of HFVs, there was a broad and strong absorption peak at 3,421–3,449 cm^−1^ which belonged to O-H stretching vibration. According to our previous research results, there were obvious -OH bands at 3,443 and 3,428 cm^−1^, which were from original HEC (Zhao et al., 2016) and FV powder (Chen et al., 2021a), respectively. The absorption peak of the mid-intensity at 2,930 cm^−1^ was caused by C-H vibration. The absorption band at 1,000–1,200 cm^−1^ in the FTIR spectrum suggested the existence of pyranose monomers (Zhang et al., 2013). In HFVs, the peaks at 1,057–1,094 cm^−1^ increase with the increase of the FV content, that is, the characteristic peak intensity HFV70 > HFV40 > HFV10. There were obvious -NH bands at 1,405–1,409 cm^−1^ in HFVs, which agreed with the reported FV polysaccharide spectrum, which had a band at 1,350–1,485 cm^−1^ that was assigned to -CH (O-CH2) flexural vibrations (Zhang et al., 2013). The absorption peak at 1,644 cm^−1^ was caused by –NH stretching vibration in amide, and this can be attributed to the fact that –NH in FV underwent an amidation reaction with -OH in HEC.

XRD patterns were established to detect the crystal structure. According to our previous research results, HEC showed a characteristic peak at 2θ = 20° (Zhao et al., 2016), while the FV sodium hydroxide extract showed two characteristic peaks at 2θ = 37 and 43°, respectively (Chen et al., 2021a). As shown in Figure 2E, HFVs showed characteristic peaks of HEC and FV sodium hydroxide extract at 2θ = 20, 37, and 43°, respectively, and another characteristic peak appeared at 2θ = 30°, presumably owing to a high glucan presence in the FV extract (Anjugam et al., 2016; Wang et al., 2017). The characteristic peak at 30° increases with the increase of the FV content. Obviously, compared with the XRD curve of the original HEC powder (Zhao et al., 2016), the peak intensity at 20° in HFV10 had almost disappeared, which confirmed that the crosslinking reaction had indeed occurred and the original ordered structure of HEC was broken. Moreover, the intensity of each characteristic peak of HFV70, HFV40, and HFV10 decreased in turn, indicating that with the decrease in the relative content of FV, the degree of crosslinking increased, and HFVs became more amorphous, which was reflected in the more compact microstructure.

#### 3.2.2 Structure and surface morphologies of the HFVs

Figure 2A shows the macro and micromorphology of cryogels with a different FV content. We first investigated the macroscopic structure. As shown in the images (Figure 2A), the color of dry HFVs gradually changed from white to yellow as the FV content increased. After wetting, it can be seen with the naked eye that the degree of expansion of HFVs increases with the increase of the FV content. The previous photographs indicate that the appearance of the HFVs depended on the FV content. To further investigate whether the FV content changed the other properties, especially surface topography and microstructure, microcomputed tomography (micro-CT) and scanning electron microscopy (SEM) were performed in subsequent experiments.

As shown in the micro-CT image (Figure 2A), all three groups of HFVs have a homogeneously distributed scaffold structure. Moreover, with the increase of the FV content, the scaffold structure gradually becomes less dense. By analyzing the mass and volume of the scaffolds, the total porosity of the scaffolds was calculated (HFV10: 73.0 ± 0.3%; HFV40: 85.1 ± 4.1%; HFV70: 95.0 ± 1.7%), and the differences among them were significantly different (Figure 2C). The results of micro-CT and porosity were also verified in SEM images (Figure 2A). The transverse sectional surface of the HFV10 presents a dense scale-like structure, so the pore size cannot be measured accurately. In HFV40, the porous structure begins to appear, but it is still relatively dense in general, with an average pore size of 80.05 ± 6.938 μm (Figure 2B). The cross-sectional surface of HFV70 looks loose and porous, and the average pore size is 151.6 ± 8.636 μm (Figure 2B), which is significantly higher than that of HFV40. At high magnification, it can be seen that the loose pores in HFV70 are interconnected. The difference in porosity and pore size of HFVs can be explained by the difference in the crosslinking degree. The relatively lowest crosslinking degree of HFV70 led to a loose and porous structure. The XRD results can support the abovementioned explanation.

Our experimental results proved that, first, by applying the freeze-drying technique, the pore size and morphological structure of the produced HFV cryogels were well controlled; second, FV can act as an effective modulator of the micromorphology of HFV cryogels. In the preparation of polymeric porous materials, the freeze-drying technique provides a well-controlled gas-phase structure for material formation. Under a low-temperature environment, the loss of volatile substances is limited, ensuring that the product is stable and favoring the formation of sponge-like solid materials (Gun’ko et al., 2013).

#### 3.2.3 Hydrophilic properties and shape memory property

We measured the water absorption capacity and water retention capacity of three groups of HFVs. As shown in Figure 4B, the water uptake ratio of HFVs increased rapidly before 0.5 h. Then, the water absorption ratio increased slowly until it reached its highest point at about 1 h. After that, the water absorption experienced a slight decrease and reached equilibrium at about 2 h, and this may be related to the dissolution and release of soluble substances (such as polysaccharides) in cryogels. We can observe that among the three groups of HFV, HFV70 has a much higher water absorption capacity than the other two groups, with a peak absorption ratio of 2,570.24%. In other words, the higher the FV content, the stronger the water absorption capacity is. It is in accordance with the results of SEM results that this open and loose porous structure helps HFV70 to absorb a large amount of liquid in a short time. In the water retention capacity assay (Figure 4C), HFV70 also had the best water retention performance as its rate of water loss was the slowest compared with the other two groups. Interestingly, we observed a water-responsive shape memory property in HFV70. As shown in Figure 4A, HFV70 could be compressed and shape-fixed after squeezing out the free water. Upon absorbing the water, it could recover to its original shape, giving a 100% recovery ratio. This can be explained by the fact that the water-responsive shape memory ability was endowed with HFV70 due to the good hydrophilicity of HEC and FV and the relatively stable cross-linked structure at the optimal ratio.

#### 3.2.4 Mechanical characterization

The mechanical properties of HFVs were tested as described as follows. The compression stress–strain curve shown in Figure 2F reveals that the increase of the FV content reduces the stiffness of HFVs. It can be explained as the increase of porosity, which is mutually confirmed with the results of micro-CT and SEM analysis in the previous section. As shown in Figures 2G–I, in tensile tests, with the increase of the FV content, the ductility and flexibility of HFV were improved. Based on those previously mentioned, HFV70 has the best comprehensive mechanical properties for wound healing *in vivo*, for its characteristics of softness, good ductility, and easy deformation are conducive to the filling and attachment of irregular wound defects.

#### 3.2.5 *In vitro* degradation kinetics

The degradation behavior is an important factor that influences the *in vivo* application of the HFVs, for cryogels will directly contact the tissue for a long duration and release bioactive components. The weight loss of the HFVs in phosphate-buffered solution (PBS) was analyzed for 14 days (Figure 4D). All the cryogels degraded with longer incubation times. The HFVs degraded sharply in the time interval of 1–3 days, which might be due to the incomplete crosslinking of FV and HEC by ECH. The HFVs showed higher weight loss as the FV content increased, and HFV70 showed the highest weight loss, which may be due to the more unassociated chains in the gel network and higher hydrolysis reaction from more water absorption. The *in vitro* degradation rate of all HFVs could meet the requirements of biomaterials for skin defect repair.

### 3.3 Cytocompatibility and hemocompatibility *in vitro*

**FIGURE 3:**
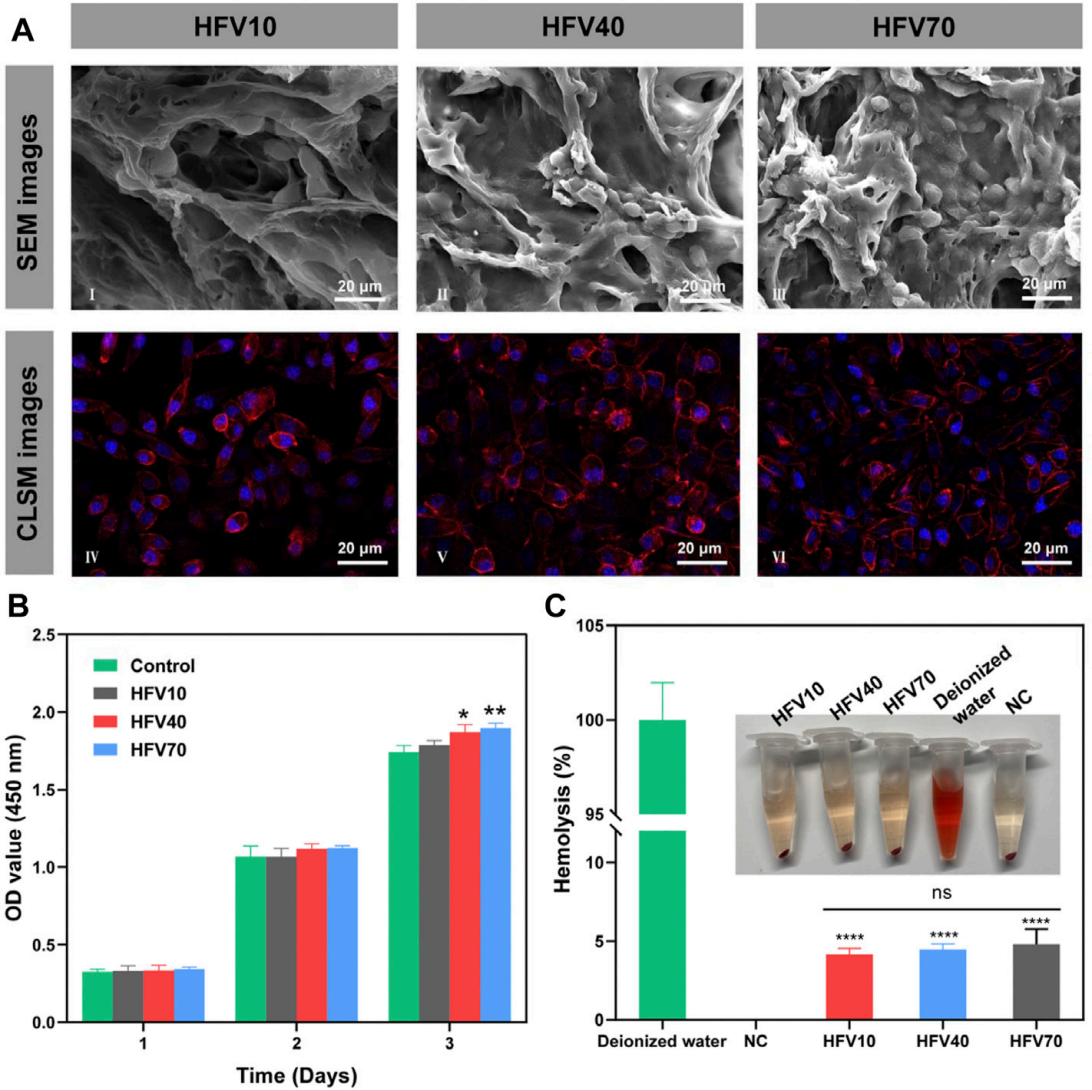
Evaluation of cell compatibility and blood compatibility of HFVs *in vitro*. **(A)** SEM (I–III) and confocal microscopic images (IV–VI) of L929 cells cultured on HFVs after 3 days. **(B)** CCK8 results of L929 cells cultured in extracts from HFVs for 1, 2, and 3 days. **(C)** Macro photographs of the hemolysis test and the corresponding hemolysis percentage. *p < 0.05; **p < 0.01; ****p < 0.0001, in comparison with the control group.

First, a CCK-8 assay was applied to determine the toxicity of HFVs. The HFV extract was used for cell culture. As shown in Figure 3B, the HFV extract had no negative effect on the growth of L929 cells. Moreover, interestingly, the extracts of HFV40 and HFV70 showed a slight promoting effect on cell proliferation on the third day. This result indicated that the toxic epichlorohydrin solution and sodium hydroxide solution used in the material preparation process were effectively washed away. In addition, this is consistent with previous research results, which showed that the extract from FV had no significant cytotoxicity to L929 cells (Chen et al., 2018; Hu et al., 2019). We speculate that the nutrient contents in FV (essential amino acids, dietary fibers, polysaccharides, and steroids) may be the contributors to the slight proliferation effect on L929 cells. To evaluate the growth state of cells on the material, L929 cells were cultured on the scaffolds for 3 days. As shown in Figure 3A, typical cell clusters and 3D growth were observed in all scaffolds. Scanning electron microscopic images showed satisfactory attachment of L929 cells to all HFVs. L929 cells revealed a good spreading morphology of the cells on the scaffolds. Among them, L929 cells adhered inside the pore wall of HFV70 were the most densely packed and exhibited a spindle-like morphology with widespread filopodia and lamellipodia, which suggests that the loose and porous morphology provided a sufficient surface area for cell attachment. Confocal microscopic images (Figure 3A) showed the best growth morphology of L929 cells on HFV70 with a spindle shape. Cell adhesion and the elongated cell morphology on porous scaffolds could be explained by the classical contact guidance theory (Xiao et al., 2021). Ma et al. (2003) suggested that microstructures with a 100–200 μm mean pore size were suitable for skin tissue engineering, and the average pore size of HFV70 is 151.6 ± 8.636 μm, which is just in this optimum range. Based on the previous experimental results, HFV70 with loose and interconnected pores provides the most convenient environment for the attachment and growth of L929 cells. In our previous two studies on biomaterials based on FV extracts, the seedbed-like FV polysaccharide–derived scaffolds apparently promoted L929 cell growth relative to the blank control (Chen et al., 2021a), and the natural FV-based nerve guidance conduit could be beneficial for the proliferation of PC-12 cells (Chen et al., 2021b). In addition, cells presented favorable adhesion, as confirmed *via* observation. Through comprehensive analysis, we can draw a conclusion that this series of biomaterials based on FV extracts all have good *in vitro* cytocompatibility and provide a native ECM-mimetic microenvironment for cells.

Hemocompatibility is also a critical factor in the performance evaluation of biomedical materials. According to the standard guideline of ASTM F 756-08, the hemolysis rate of biomedical materials that contact blood should be less than 5% (Wu et al., 2021). The hemolysis assay (Figure 3C) showed that the hemolysis rates of all HFVs were below the detection line of biomedical hemolysis rates (<5%), which indicated that all cryogel samples could not elicit severe hemolysis.

### 3.4 Antibacterial property *in vitro*

Wound infection is one of the main obstacles to wound healing, so modern dressings are designed to be endowed with the anti-infection function. The antibacterial assays of the HFVs were tested against *Escherichia coli* or Gram-positive *Staphylococcus aureus via* the antibacterial disc susceptibility method, as shown in Figure 5C. Then, the zone of inhibition (ZOI) describing the area of the bacteria-free zone was recorded and analyzed (Figures 5D, E). HFVs exhibited different degrees of activity against *E. coli* and *S. aureus* when compared with the negative control (blank filter paper). The areas of ZOI against *E. coli* of HFV10, HFV40, and HFV70 were 0.736667 ± 0.204975, 1.774 ± 0.197226, and 2.861333 ± 0.106562 cm^2^, respectively (Figure 5D), while the areas of ZOI against *S. aureus* of HFV10, HFV40, and HFV70 were 0.6703 ± 0.154088, 1.613033 ± 0.028614, and 3.138633 ± 0.140466 cm^2^, respectively (Figure 5E). The antibacterial properties of HFVs may be due to the effects of bioactive components of FV. In the previous literature, reports on the occurrence of antimicrobials in FV are well documented. It was reported that methanol (Karaman et al., 2010), chloroform (Karaman et al., 2010), or hot water extracts (Dong et al., 2017) of *Flammulina velutipes* display antibacterial effects. Enokipodins are a group of α-cuparene-type sesquiterpenoids that have been isolated from FV and are known to be major constituents responsible for FV’s antimicrobial activities (Ishikawa et al., 2001; Wang et al., 2012a; Wang et al., 2012b). In our previous study, seedbed-like FV polysaccharide–derived scaffolds (FPDSs) were prepared by the frozen sectioning process for wound healing, and it was proved that sodium hydroxide treatment can increase the antibacterial activity of the original FV scaffolds (Chen et al., 2021a). According to our experimental results, the preparation process of HFVs effectively retains the antibacterial substances in FV and ensures their effective release. Furthermore, the impact of the HFVs on the growth of the examined bacteria was enhanced distinctly with the increase of the FV concentrations.

### 3.5 Antioxidant property *in vitro*

Oxidative stress at the wound site is one of the main causes of inflammation. A great amount of reactive oxygen species (ROS) is produced in the process, which delays the wound healing process. The stable 1,1-diphenyl-2-picrylhydrazyl (DPPH) free radical is the most commonly used reagent to test the ROS scavenging ability of materials, the scavenging of which is detected by measuring absorbance at 517 nm. As shown in Figures 5F, G, the DPPH scavenging activity was positively correlated with the FV content. The DPPH scavenging rate of HFV70 was as high as 74.55%. The experimental results are consistent with the previous research results. FV has an excellent antioxidant capacity, which may be due to its compounds, polyphenols. Polyphenols, derived from the metabolic pathway of phenyl propane, contain multiple hydroxyl groups and at least two phenolic rings and could eliminate excessive free radicals by joining the reactive oxygen species and active nitrogen (Ma et al., 2021). Hence, HFV70 with excellent antioxidant properties presents considerable potential in further wound healing applications.

### 3.6 Angiogenic property *in vitro*

According to the Matrigel experiment (Figure 5A), the total length of the vessel-like tubes formed by HUVEC cells of the three groups of the HFV extract increased compared to the control group, and the angiogenic effect of the HFV70 group was the most significant, which suggests the trophic effects of the FV. The mechanism of HFV70-induced neovascularization will be further discussed in future animal experiments.

### 3.7 *In vivo* hemostatic behavior

**FIGURE 4:**
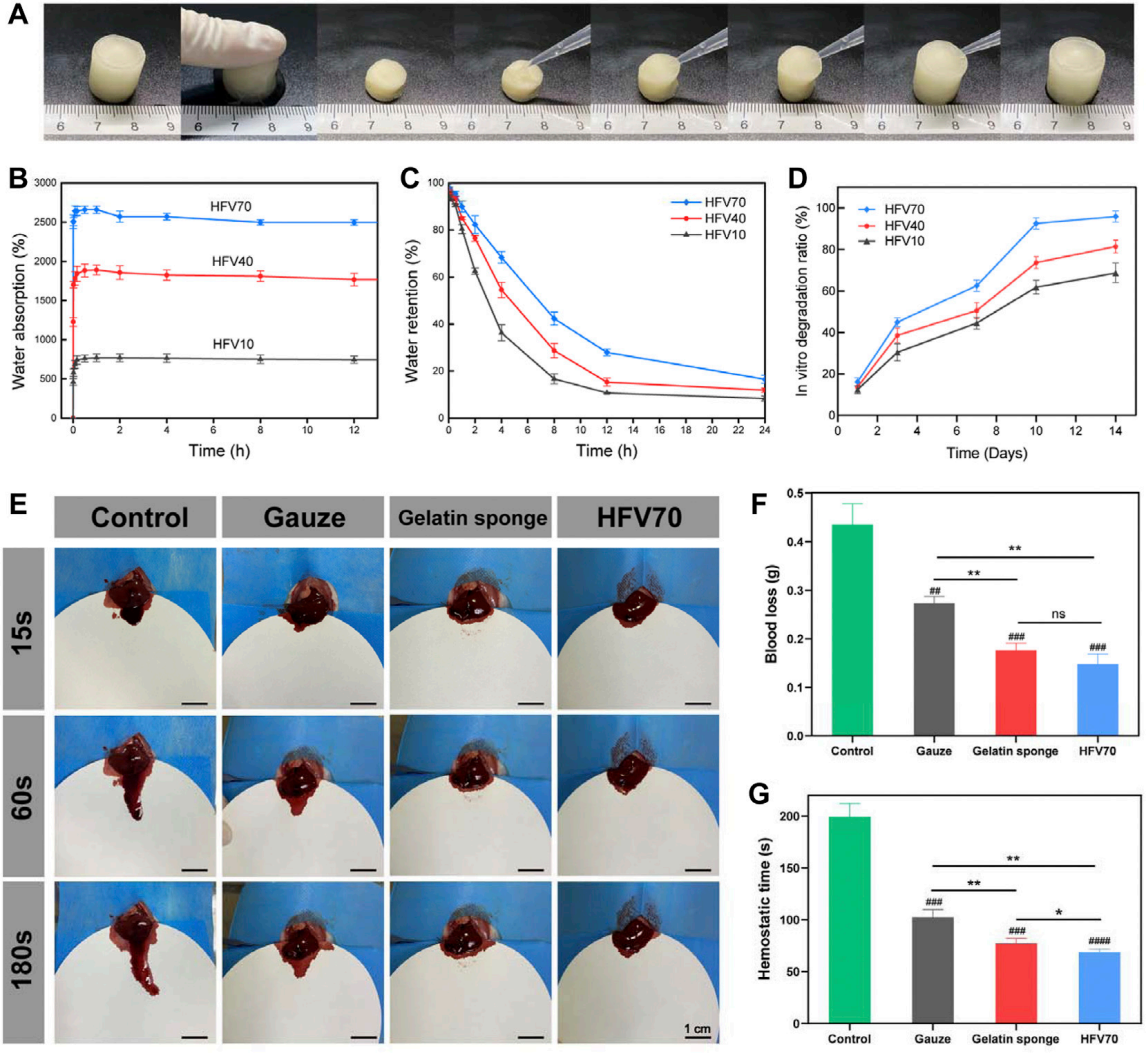
Shape memory performance, water absorption and retention test, and hemostasis in the normal rat liver perforation wound model. **(A)** Macro photographs of the water-triggered shape recovery of HFV70. **(B)** Water absorption of HFVs. **(C)** Water retention of HFVs. **(D)** Dependence of the *in vitro* degradation ratio of HFVs soaked in PBS on soaking time. **(E)** Photographs of the hemostatic effect of the gauze, gelatin sponge, and HFV70. **(F)** Blood loss from the liver incision. **(G)** Hemostatic time of different treatment groups. *p < 0.05; **p < 0.01; ***p < 0.001; ****p < 0.0001, in comparison with the control group.

**FIGURE 5:**
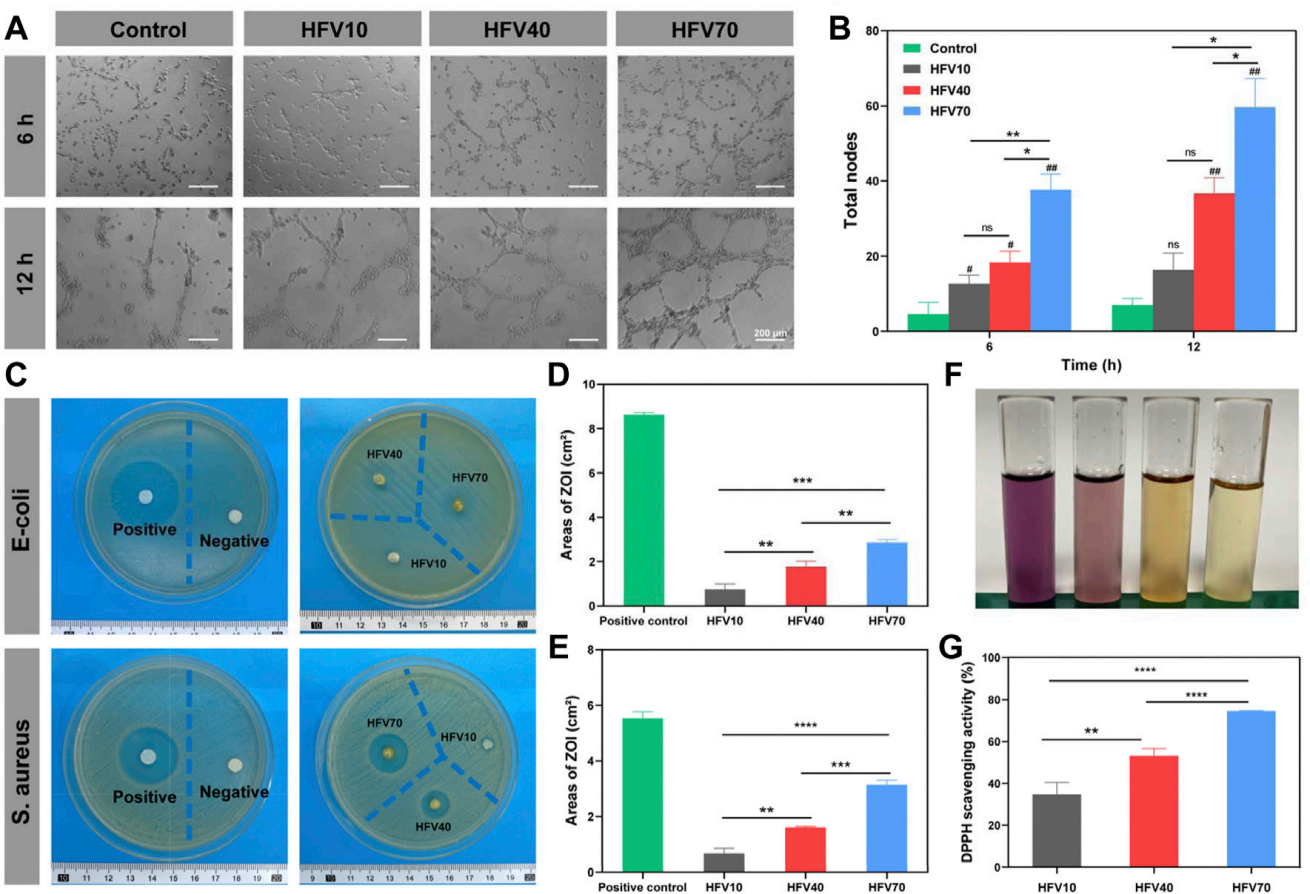
Evaluation of angiogenesis and antibacterial and antioxidant properties of HFVs *in vitro*. **(A)** Images of HUVEC tube formation. **(B)** Quantified results of the total tube nodes of HUVEC tube formation. **(C)** Inhibition zone images of *E. coli* and *S. aureus* on agar plates after being treated with samples. **(D)** Areas of ZOI against *E. coli* by the HFVs. **(E)** Areas of ZOI against *S. aureus* by HFVs. **(F)** Macro photographs of the DPPH scavenging test. **(G)** DPPH scavenging rates. *p < 0.05; **p < 0.01; ***p < 0.001; ****p < 0.0001, in comparison with the control group.

Porous and fibrous micro-structured scaffolds are newly emerging hemostatic agents during severe intraoperative or extra-operative bleeding as they can easily pressurize the interior of the tissue defect and be removed from the bleeding site (Sultana et al., 2021). The ability of HFV70 to address noncompressible bleeding was evaluated in a rat liver defect model (Figure 4E). Compared with the blank control group, the HFV70 group significantly reduced the blood loss (P<0.001) (Figure 5E) from 0.435 g to 0.148 g HFV70 was comparable to a commercial gelatin sponge (0.177 g) in the control effect of blood loss. In terms of hemostasis time control, HFV70 was even slightly better than gelatin sponge (*p* < 0.05). Traditional hemostatic materials are usually difficult to deal with in cases of incompressible bleeding (Huang et al., 2021). However, HFV70 with shape memory performance and a high swelling ratio could seal the wound well because it expands and locally concentrates the blood after blood absorption. The previous results indicate that the HFV70 cryogel has a good hemostatic ability and can cope with noncompressible hemorrhage.

### 3.8 Subcutaneous defect filling and repair evaluation

**FIGURE 6:**
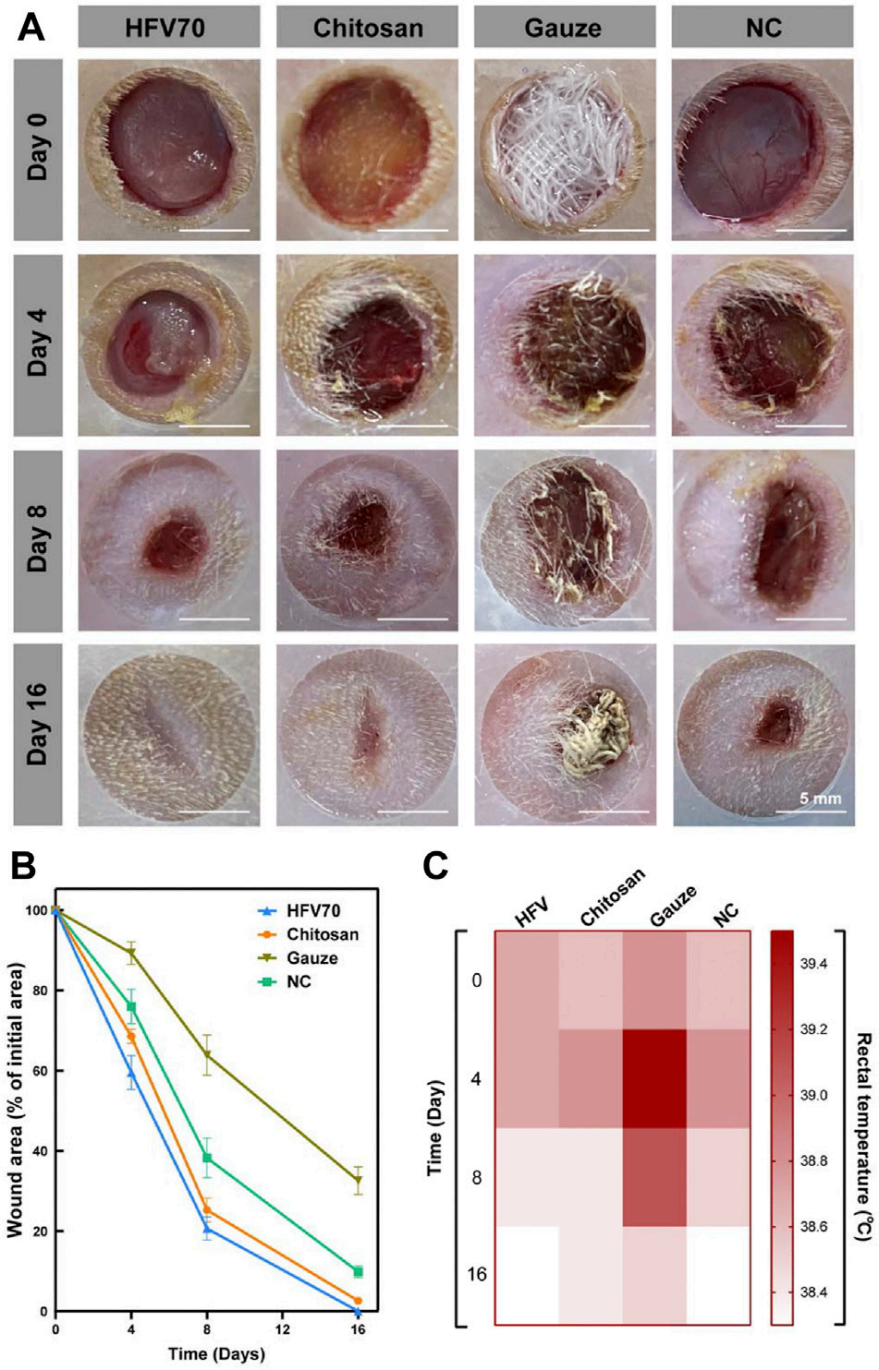
Treatment effect evaluation in the rat full-thickness skin defect model. **(A)** Images of initial wounds covered with different dressings and the subsequent images of the wounds at different time points (4, 8, and 16 days). **(B)** Wound area changes in each group. **(C)** Body temperature changes in each group.

The ability of HFV70 to promote wound healing was studied in a rat full-thickness skin defect model. Figures 6A,B exhibited the photographs and wound size treated with HFV70, chitosan, gauze, and blank control group for 0, 4, 8, and 16 days. The wound contraction of the HFV70 cryogel group had the advantage over other groups. The wound sizes of the gauze group, NC group, chitosan group, and HFV70 group decreased in sequence at the same time point. In the early stage of wound healing (<8 days), the HFV70 group showed a moister wound surface. After 16 days of treatment with HFV70, the wound completely healed and there was little scar formation. We also measured the body temperature of rats at various time points. As shown in Figure 6C, it was found that the mean body temperature of the HFV70 group was relatively lower than that of other groups, especially the gauze group, and the relatively lower body temperature reflects a less severe inflammatory condition. We made a preliminary analysis of the reasons for the improvement of wound healing by HFV70 as follows: first, HFV70 has good biocompatibility and anti-inflammatory and antibacterial properties; second, HFV70 is flexible and has shape memory property, which makes it accommodate the wound well; third, its porous microscopic characteristics provide the necessary supporting structure for cell growth, and the interconnected pores are conducive to the exchange of nutrients and waste metabolites.

In addition, the high water retention of HFV70 provides a moisture-suitable environment during the early stage of wound healing.

### 3.9 Histological analysis

**FIGURE 7:**
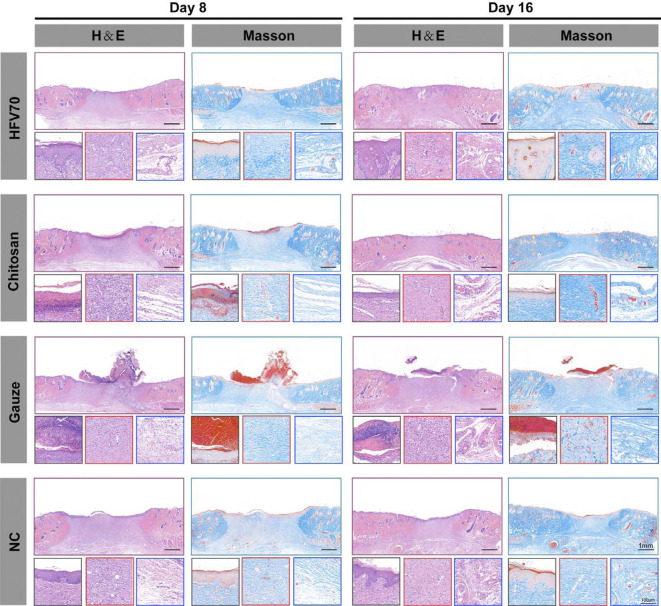
H&E and Masson’s staining images of wound areas in each group and detailed images corresponding to the epidermis (black square border), dermis (red square border), and subdermal tissues (blue square border).

The wound healing of the dermal defect was assessed by observing tissue sections. H&E and Masson staining were performed on days 8 and 16 for histological analysis. As shown in Figure 7A, at the same time point, the HFV70 group had the highest degree of angiogenesis and the densest collagen reconstruction compared with the other three groups. At the same time point, the HFV70 group showed the best effect of re-epithelialization and re-vascularization and less inflammatory cell infiltration. On day 16, the wound in the HFV70 group was completely re-epithelialized, and the granulation tissue was thickened and stratified. The structure of the new skin was similar to that of the surrounding normal skin. The collagen fibers were arranged neatly, and the hair follicles and glands were formed.

**FIGURE 8:**
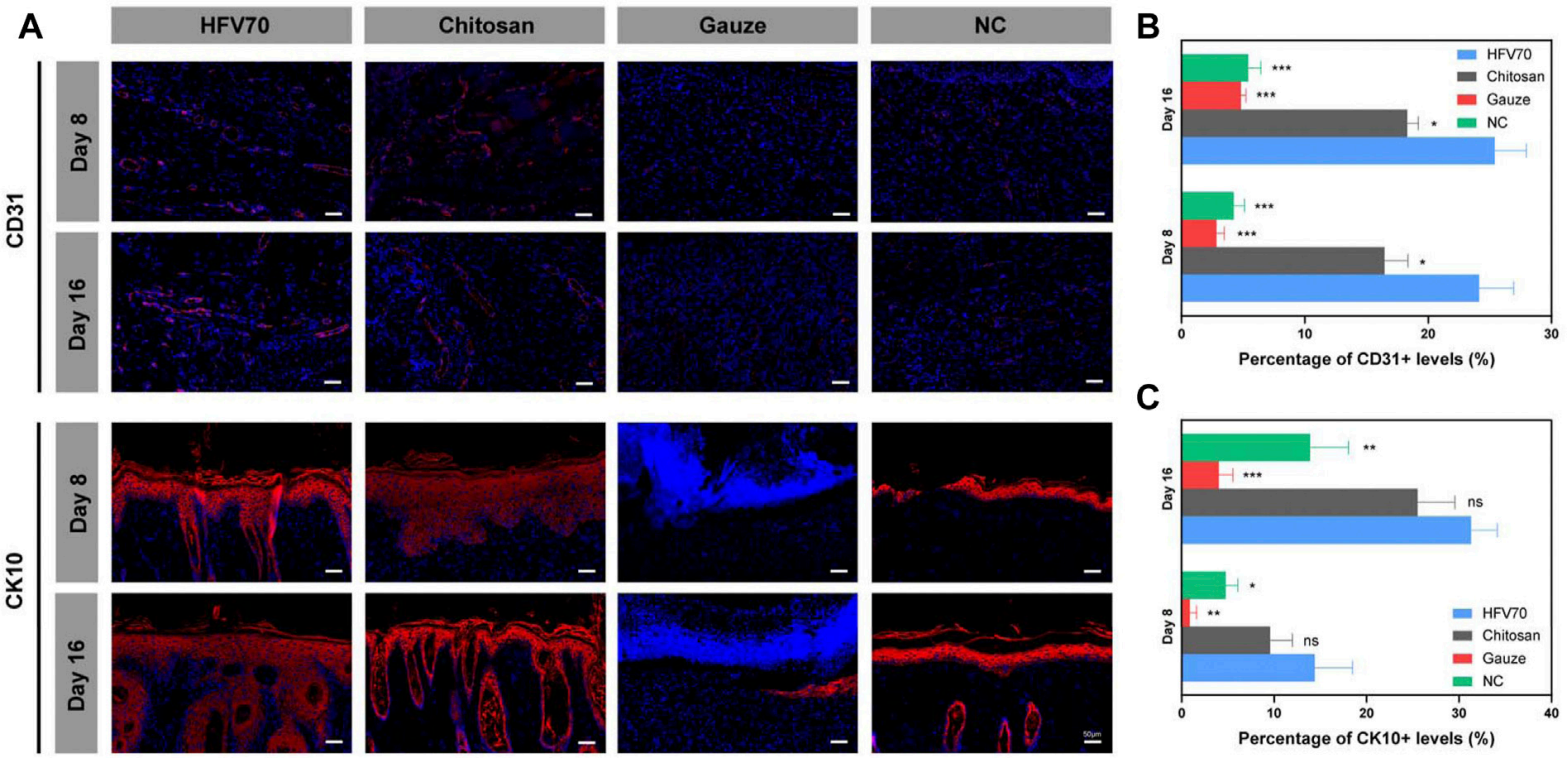
Immunofluorescence analysis of wound tissues. **(A)** Representative images of regenerated skin tissues stained with anti-CD31, anti-CK10 (red), and DAPI (blue). **(B)** Analysis of positive staining ratios of CD31 in the healing wound sections. **(C)** Analysis of positive staining ratios of CK10 in the healing wound sections. *p < 0.05; **p < 0.01; ***p < 0.001; *comparing to the HFV70 group.

Immunofluorescence methods were used to evaluate the effects of different treatments on angiogenesis and re-epithelialization of skin defects. As shown in Figure 8A, the cluster of differentiation (CD31) and cytokeratin 10 (CK10) were stained red in the wound sections. After DAPI staining, the cell nuclei were shown to be blue and complete. The HFV70 group displayed more CD31-positive stained vessels on day 8 and day 16 than those in the other three groups, respectively. The immunofluorescence staining of CK10 showed that the newly formed epidermis of the HFV70 group and chitosan group were structurally intact with more layers. The epidermal structure of the NC group was relatively intact but with a low number of layers. The gauze group showed no intact epidermis-like structure.

Taken together, the results of histological studies demonstrate good biocompatibility and anti-inflammatory and pro-angiogenic activities of HFV70 *in vivo*. HFV70 can effectively promote skin defect re-epithelialization, and its effect is no less than that of commercial chitosan dressings.

## 4 Conclusion

In summary, we have presented an economical, green, and simple method to fabricate a series of cryogels using FV and HEC as main components. The performance of the HFVs was evaluated to determine the optimal ratio of FV and HEC to fulfill the essential biological and physical requirements for the treatment of full-thickness skin defects. The results showed that HFV70 with a high content of FV (70%) exhibited an appropriate micromorphology, satisfactory ductility, highly hydrophilic properties, and a desirable degradation rate for tissue engineering. The high water absorption and shape memory properties of HFV70 allow it to effectively fill tissue defects and play a role in rapid hemostasis. The results of the *in vitro* study demonstrated that HFV70 yielded a higher number of attached cells and more favorable growing conditions for L929 cells, and it also exerted antibacterial, antioxidant, and pro-angiogenic activities. More importantly, HFV70 provided a moist microenvironment and released bioactive substances, which promoted the vascularization and re-epithelialization of wounds, as well as played an anti-inflammatory role when applied to the rat full-thickness skin defect model. This study not only provided a novel and multifunctional approach for skin repair and rapid hemostasis but also shed new light on the new strategies of the application of natural polymers for skin regeneration.

## Data Availability

The original contributions presented in the study are included in the article/Supplementary Material; further inquiries can be directed to the corresponding authors.
