# Supplementary material for: Biocompatible and antibacterial Flammulina velutipes-based natural hybrid cryogel to treat noncompressible hemorrhages and skin defects
Source: Front Bioeng Biotechnol. 2022 Oct 11;10:960407. doi: 10.3389/fbioe.2022.960407 (PMC9593062; doi:10.3389/fbioe.2022.960407)
Supplement: Supplementary file 1 [file DataSheet1.docx]

Supplementary Material


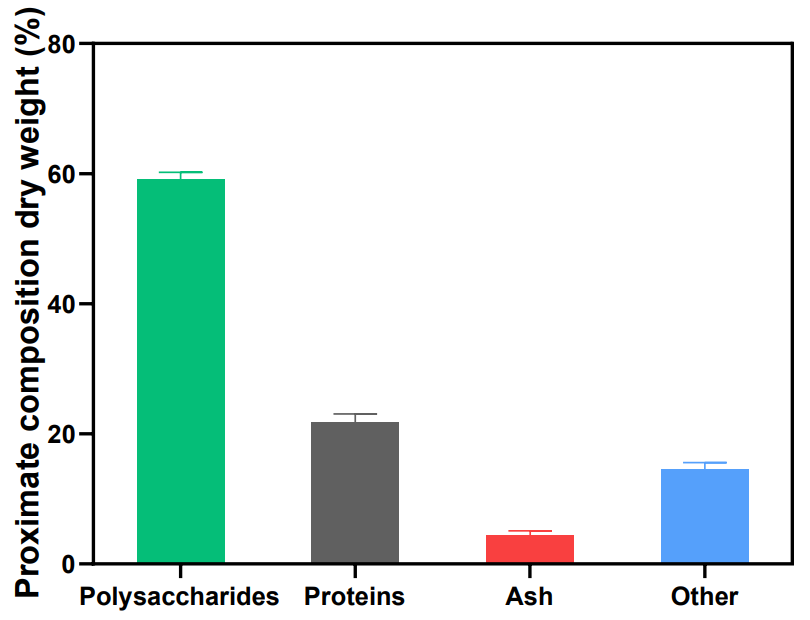


**Supplementary Figure 1.** Biochemical composition (%) of Flammulina velutipes extracts.
